# The Interplay between Drivers of Erythropoiesis and Iron Homeostasis in Rare Hereditary Anemias: Tipping the Balance

**DOI:** 10.3390/ijms22042204

**Published:** 2021-02-23

**Authors:** Simon Grootendorst, Jonathan de Wilde, Birgit van Dooijeweert, Annelies van Vuren, Wouter van Solinge, Roger Schutgens, Richard van Wijk, Marije Bartels

**Affiliations:** 1 Department of Clinical Chemistry and Haematology, University Medical Center Utrecht, 3584 CX Utrecht, The Netherlands; S.T.Grootendorst-3@umcutrecht.nl (S.G.); j.r.a.dewilde@students.uu.nl (J.d.W.); B.vanDooijeweert-3@umcutrecht.nl (B.v.D.); W.W.vanSolinge@umcutrecht.nl (W.v.S.); R.vanWijk@umcutrecht.nl (R.v.W.); 2 Van Creveldkliniek, University Medical Center Utrecht, 3584 CX Utrecht, The Netherlands; A.J.vanVuren@umcutrecht.nl (A.v.V.); R.Schutgens@umcutrecht.nl (R.S.)

**Keywords:** erythropoiesis, iron metabolism, iron overload, ineffective erythropoiesis

## Abstract

Rare hereditary anemias (RHA) represent a group of disorders characterized by either impaired production of erythrocytes or decreased survival (i.e., hemolysis). In RHA, the regulation of iron metabolism and erythropoiesis is often disturbed, leading to iron overload or worsening of chronic anemia due to unavailability of iron for erythropoiesis. Whereas iron overload generally is a well-recognized complication in patients requiring regular blood transfusions, it is also a significant problem in a large proportion of patients with RHA that are not transfusion dependent. This indicates that RHA share disease-specific defects in erythroid development that are linked to intrinsic defects in iron metabolism. In this review, we discuss the key regulators involved in the interplay between iron and erythropoiesis and their importance in the spectrum of RHA.

## 1. Introduction

Rare hereditary anemias (RHA) are a heterogenous group of diseases caused by genetic defects, resulting in impaired production or increased destruction of red blood cells (RBCs). The incidence of hereditary anemias varies, with hereditary spherocytosis, thalassemia and sickle cell disease being the more common, while Diamond-Blackfan anemia, congenital dyserythropoietic anemia and pyruvate kinase deficiency are among the rarer anemias [1,2,3,4]. In RHA, iron overload (IO) is a common complication, which can lead to severe organ damage, including liver fibrosis, heart failure and endocrine dysfunction [5]. This occurs universally in transfusion-dependent patients, but is also common in patients with RHA who are non-transfusion-dependent [6]. Most knowledge of IO is based on hereditary hemochromatosis (HH) patients, where iron loading occurs due to mutations in genes involved in iron metabolism. [7]. Although the pathophysiology of transfusional IO is well-understood, this is not the case for non-transfusion-dependent anemia [8]. There has been much debate concerning the interplay between erythropoiesis and iron homeostasis, yet disease-specific regulators of iron metabolism in these anemias are largely unknown. Here we aim to give an overview of disease-specific and common regulators of iron metabolism with respect to disease mechanisms in RHA. This will increase our understanding of the important regulators of iron metabolism, and elucidate what still needs to be investigated to further develop therapeutic interventions. 

### Iron Metabolism in Erythropoiesis

**Figure 1:**
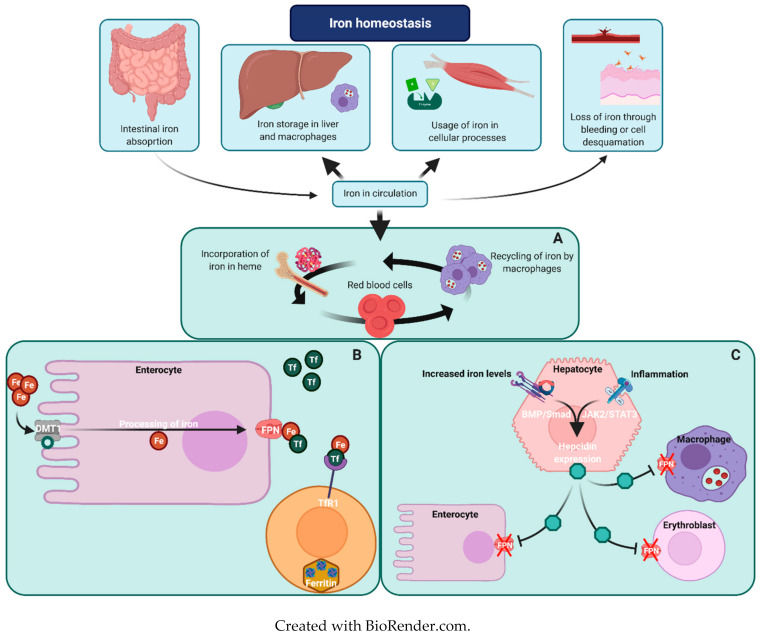
Simplified overview of iron homeostasis. Thickness of arrows reflects amount of iron per compartment. In humans, daily absorption of iron, as well as iron loss, is minimal. Most iron is either stored, used for cellular processes or is present in the erythroid compartment. (**A**) Iron recycling within the erythroid compartment. Iron is incorporated in hemoglobin and recycling of iron occurs due to splenic clearance of senescent red blood cells. (**B**) Iron absorption, transport and storage. Enteral absorption is facilitated by divalent metal transporter 1 (DMT1). Iron is processed and secreted to the circulation via ferroportin (FPN), the carrier protein through which stored iron is also secreted from within macrophages and hepatocytes. In the circulation iron binds to transferrin (Tf) and cellular uptake occurs upon binding of Tf to transferrin receptor 1 (TfR1). Iron is primarily stored as ferritin within cells. (**C**) Hepcidin-ferroportin axis. Hepcidin expression is predominantly induced by the bone morphogenetic protein (BMP)/Smad signaling pathway, upon increased serum iron levels. Alternatively, the Janus kinase 2/Signal Transducer and Activator of Transcription 3 (JAK2/STAT3) pathway is activated upon inflammation through interleukin-6 (IL-6). Hepcidin blocks FPN-dependent iron export, and induces FPN degradation, resulting in a limitation of iron absorption and iron efflux from body iron storages [16].

Iron plays an important role in a large variety of cellular processes, yet excess iron can lead to generation of reactive oxygen species which may damage cellular components [9]. The regulation of normal iron homeostasis has been extensively reviewed elsewhere and is briefly summarized in Figure 1 [10,11,12]. Organs that are particularly vulnerable for IO are the liver, heart and pancreas. Iron excess in these organs may lead to liver cirrhosis, heart failure and diabetes mellitus [8,13]. Assessment of iron status can be challenging. Historically, ferritin has been used as a parameter to screen for IO; however, it has been demonstrated that ferritin levels may be relatively low in RHA while patients suffer from severe IO. Transferrin saturation (TSAT), magnetic resonance imaging (MRI) and levels of non-transferrin bound iron (NTBI) are more adequate diagnostic tools for the assessment of iron status in RHA (Table 1) [6,13,14,15].

**Figure 2:**
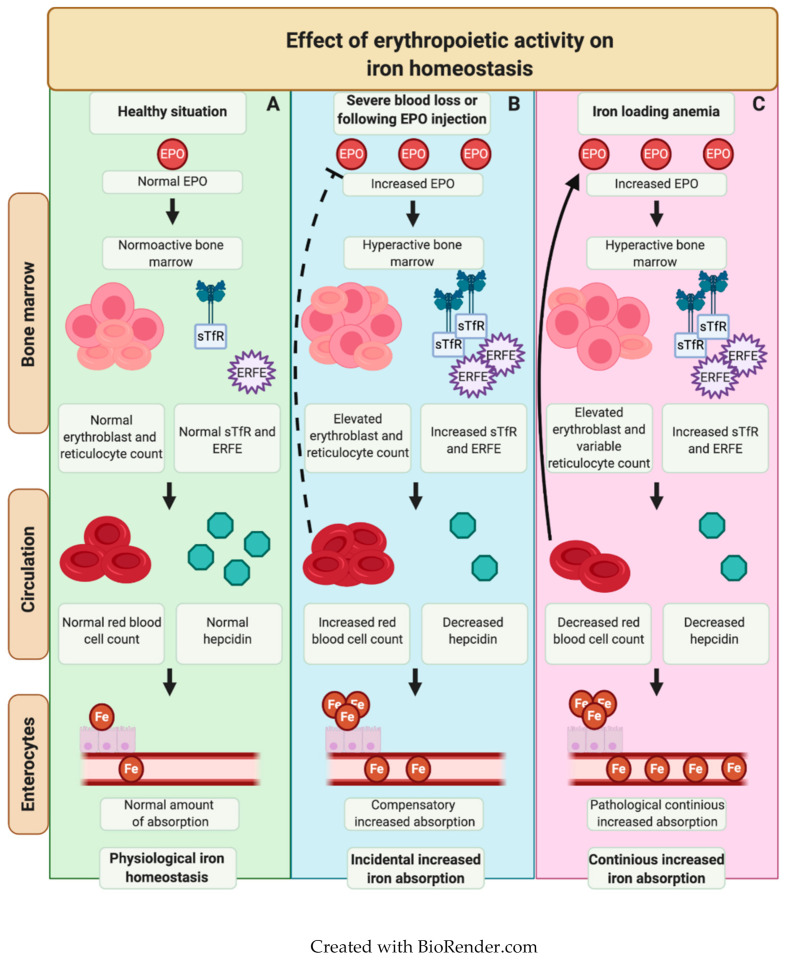
Simplified overview of connecting factors in erythropoiesis and iron metabolism. (**A**) Healthy situation. Normal production of red blood cells (RBCs) to prevent tissue hypoxia, erythropoietin (EPO) levels are low. erythroferrone (ERFE) levels are normal, little impact on iron metabolism. (**B**) Acute anemia or EPO injection. Low levels of oxygen lead to increased EPO levels. EPO stimulates erythroblast proliferation and maturation and subsequently upregulation of soluble transferrin receptor 1 (sTfr1) and ERFE levels. Hepcidin levels are suppressed via ERFE, allowing increased iron uptake, followed by incorporation in hemoglobin in erythroblasts. This results in an increase in RBCs and compensation for the anemia. (**C**) Iron loading anemia. This is characterized by ineffective erythropoiesis, resulting in suboptimal compensation for anemia, leading to a constitutively hyperactive bone marrow and a persistent increase in EPO and sTfR levels, with or without high reticulocyte numbers. Iron levels are sufficient to cover for erythroid demand. Hepcidin levels are persistently low, partially due to high ERFE levels, leading to iron overload.

Erythropoiesis has a great demand for iron, which is mainly incorporated in red blood cell hemoglobin. Erythroblasts highly express Transferrin receptor (Tfr1), and its expression is further upregulated during increased erythroid drive [18]. During erythroblast maturation, Tfr1 is cleaved off and can be detected in the circulation as soluble transferrin receptor 1 (sTfr1). Consequently, increased levels of sTfr1 indicate an increase in erythropoietic drive [19,20,21]. Senescent erythrocytes are removed in the spleen, where iron is recycled by macrophages. Due to the close connection between iron and erythropoiesis, it was hypothesized that hepcidin expression may be regulated by factors involved in erythropoiesis, such as erythropoietin (EPO), a hormone produced by kidney cells upon hypoxia, which is the main stimulant for erythroblast proliferation and survival [22]. In the search for an erythroid regulator, erythroferrone (ERFE) was identified in mice who underwent phlebotomy or EPO administration, which is known to result in suppression of hepcidin levels [23,24,25,26]. ERFE is a member of the C1q/tumor necrosis factor (TNF)-related protein family, encoded by the gene *Fam132b,* and is also known as myonectin or CTRP15 [27]. It is secreted by erythroblasts upon increased erythropoietic activity. Increased ERFE expression was observed following EPO receptor binding, inducing activation of the JAK2/STAT5 signaling pathway [23,26]. Upon secretion, ERFE acts as a ligand trap for BMPs, resulting in inhibition of BMP signaling and subsequent suppression of hepcidin transcription [28]. Hence, following blood loss or during chronic anemia, hypoxic conditions induce increased EPO levels, which stimulate ERFE expression by erythroblasts and consequently downregulate hepcidin production to provide extra iron supply to support erythropoiesis. Especially during ineffective erythropoiesis (a state of increased erythroid drive in the bone marrow with premature death of erythroblasts, resulting in a decreased erythroid output), ERFE levels are greatly increased, which is thought to be caused by the increased number of erythroblasts [29]. Another protein which may be increased in RHA is Growth and Differentiation Factor 15 (GDF15). GDF15 is a member of the TGFß superfamily of proteins produced by late-stage erythroblasts [30,31]. While GDF15 is not crucial during steady-state erythropoiesis, it plays a potential role in stress-erythropoiesis, as has primarily been demonstrated in mouse models [30,32,33]. However, following the findings that GDF15 deficiency did not affect hepcidin suppression in phlebotomized mice, only extremely elevated levels of GDF15 can suppress hepcidin levels ex vivo, and normal GDF15 levels have been detected in patients with non-transfusional IO, it is likely that GDF15 is primarily a marker for ineffective erythropoiesis and not a general regulator of iron homeostasis in RHA [31,33,34,35,36]. An overview of the interplay between erythropoiesis and iron metabolism is provided in Figure 2. 

## 2. Regulation of Iron Overload in RHA 

Beta-thalassemia (β-thalassemia) is a hemoglobinopathy caused by a pathogenic mutation in one or both beta-globin genes (*HBB*) and is characterized by hemolytic anemia and ineffective erythropoiesis [37]. Homozygous patients are categorized as non-transfusion-dependent thalassemia patients (NTDT, previously beta-thalassemia intermedia) or transfusion dependent thalassemia patients (TDT, previously beta-thalassemia major). It is well-known that NTDT patients are prone to developing severe IO, which has been attributed to ineffective erythropoiesis, and can be seen in the absence of high ferritin levels (ferritin <800ng/mL) [38,39,40]. The mechanism connecting erythropoiesis and the dysregulated iron metabolism in NTDT has long been unknown, with the discovery of ERFE being a breakthrough in the understanding of this mechanism [26]. Compensatory increased erythropoietic activity leads to drastically elevated ERFE levels in both NTDT patients and beta-thalassemic mice, especially due to ongoing ineffective erythropoiesis, which is reflected by the increased levels of GDF15 in patients [23,41,42]. Based on this, ERFE is currently being investigated as a potential pharmacological target in NTDT [28,43]. Moreover, the increased erythropoietic drive may lead reticulocytosis and increased sTfR levels [40,44]. Due to ongoing hemolysis and insufficient compensation of anemia, EPO levels remain high, further stimulating erythroid output and high ERFE expression. Based on this knowledge, it seems conceivable that ERFE levels can be estimated based on parameters of erythropoietic activity, including EPO, reticulocyte count and levels of sTfR. This can potentially be extrapolated to other RHA characterized by dysregulated iron metabolism and increased or ineffective erythropoiesis, and will contribute to our understanding of iron metabolism in other RHA.

Pyruvate kinase deficiency (PKD) is a congenital glycolytic enzyme defect, caused by mutations in the Liver and Red blood cell Pyruvate kinase (*PKLR*) gene, and is characterized by chronic hemolytic anemia [45,46]. IO is a serious complication in most patients suffering from both transfusion-dependent and non-transfusion-dependent PKD, which was illustrated in the Pyruvate Kinase Deficiency National History Study, reporting high serum ferritin levels (>1000 ng/mL) in 38% of non-regularly transfused patients, and elevated liver iron content (LIC) values (>3 mg/g dry weight) in 82% in this group [6,45,47]. In addition, PKD patients with relatively low ferritin levels had significant IO reflected by a 90% sensitivity for LIC>3 g/g in patients with serum ferritin levels > 500 ng/mL [6]. It has been demonstrated that hepcidin levels in PKD are suppressed [48]. Hemolytic anemia in PKD is accompanied by elevated EPO levels and compensatory reticulocytosis which is, paradoxically, further increased after splenectomy [49]. It has recently been demonstrated that EPO and mean ERFE levels were elevated in the majority of PKD patients compared to healthy controls. Of these 23 patients, six (6/23) were treated with regular transfusions and fifteen (15/23) patients were treated with splenectomy. Although hepcidin values were unavailable in this study, mean ferritin levels were 521 ng/mL (range of 225–781) and 36% of the patients received iron chelation therapy [42]. Combining this with the elevated ERFE levels, it is conceivable that the increased erythropoietic drive in PKD results in hepcidin suppression, similar to NTDT. Previous studies have demonstrated that GDF-15 was only moderately increased, suggesting a lower degree of ineffective erythropoiesis in PKD [31,50].

In alpha-thalassemia (α-thalassemia), hemolytic anemia is the result of mutations, mainly deletions, affecting one or more of the four genes encoding for alpha-globin chains (*HBA1* and *HBA2*) of hemoglobin [37,51]. Depending on the number of affected genes, alpha-thalassemia may be categorized into carrier, alpha-thalassemia minor, HbH disease and Bart’s hydrops fetalis. Patients with HbH disease suffer from symptoms typical for hemolytic anemia and are also more prone to develop IO, which is also the case in non-transfusion-dependent patients, although this has been studied less extensively than in NTDT [52,53,54]. Interestingly, in a study that compared patients with (non- or sporadically-transfused) HbH disease to NTDT, several major differences were observed. While EPO levels were relatively high and sTfR levels were significantly increased in HbH disease, indicating that erythropoiesis is increased as a compensation for chronic hemolysis, this was not accompanied by increased levels of GDF15. In addition, reticulocyte levels were comparable between NTDT and HbH disease, and on average were even higher in HbH disease. Despite median ferritin levels within the normal range, median LIC levels were 5.1 mg/g (compared to 7.0 in NTDT), indicating significant IO. Hepcidin levels were normal, yet inappropriately low in the context of total body iron [55]. Whereas little is known about ERFE levels in HbH disease, based on these findings it is conceivable that ERFE levels are not drastically increased, since hepcidin levels are relatively normal. Altogether, these data suggest that (moderately) increased erythropoiesis, and low levels of ineffective erythropoiesis in HbH disease (reflected by relatively normal EPO and GDF15 levels, and reticulocytosis), are associated with IO, albeit less severely than in NTDT.

Congenital dyserythropoietic anemia (CDA) is a group of hereditary anemias characterized by a defect in proliferation and differentiation of erythroid precursors (dyserythropoiesis). Patients may present with hyporegenerative anemia, typical morphological abnormalities and hemolysis [56,57,58]. Based on underlying genetic defects, CDA has been classified into CDA type I, II, III and IV, as well as several minor subtypes. CDA I and II are the most prevalent types [58,59] and although there are similarities in the biological and clinical features, these types are considered as different entities. In CDA type I, predominantly caused by biallelic mutations in the *CDAN1* gene (Codanin 1), chronic anemia is accompanied by hepatosplenomegaly, jaundice and secondary complications, including pulmonary hypertension and IO [56,57,60,61,62]. Two small case series have described iron parameters in CDA type I patients, demonstrating serum ferritin levels above 500 ng/mL (range of 604–2217) in nine out of 11 (9/11, 82%) non-transfusion-dependent patients, and elevated LIC values in three of four (3/4, 75%) non-transfusion-dependent patients (LIC > 3 mg/g (range of 9.94–17.3)), respectively [63,64]. In CDA type I, it has been demonstrated that serum EPO and GDF15 levels are increased, suggesting ineffective erythropoiesis [36,63]. Whereas mean hepcidin/ferritin ratios were significantly lower in CDA type I patients, mean hepcidin levels were not significantly decreased, suggesting that while iron loading might occur, it is not as severe as in NTDT, where hepcidin levels are undetectable [63]. This can potentially be explained by a lower degree of ineffective erythropoiesis and subsequently lower levels of ERFE in CDA type I compared to NTDT. However, ERFE levels have not been structurally measured in CDA type I, so also other, yet unknown factors may be involved.

CDA type II is the most frequently occurring type of CDA. It results from biallelic mutations in the *SEC23B* gene, encoding for SEC23B protein, which plays a role in the cellular secretory pathway [65,66,67]. CDA type II has a large overlap in clinical presentation with CDA type I, including IO [57,58,68]. In a retrospective study of 205 patients, the mean ferritin levels in the entire cohort and in non-transfusion-dependent patients were 464.8 ± 55.9 ng/mL and 282.2 ± 36.7 ng/mL, respectively, demonstrating a general tendency to develop IO [68]. In response to anemia, CDA type II patients have elevated EPO levels and show erythroid hyperplasia in the bone marrow, with reticulocyte counts within the normal range, yet remaining relatively low [68,69]. GDF-15 levels are increased, indicating ineffective erythropoiesis [70]. In a study that compared non-transfusion-dependent CDA type II patients with NTDT patients, it was shown that ERFE levels were comparable and significantly elevated [71]. Furthermore, while serum ferritin and TSAT were not significantly different in CDA type II (compared to NTDT), remarkably, reticulocyte counts and the bone marrow responsive index (BMRI) were higher in CDA type II, while soluble transferrin-receptor levels were lower [68]. Altogether, this suggests that ineffective erythropoiesis is more severe in NTDT than in CDA type II. In addition, when CDA type II patients were divided into two groups based on their ERFE level, it was shown that higher ERFE levels correlated with significantly higher soluble transferrin-receptor and EPO levels, while hepcidin and hemoglobin levels were significantly lower. Interestingly, there was no significant difference between serum iron parameters in both groups [71]. In conclusion, IO in CDA type II may be attributed to ineffective erythropoiesis. Since iron parameters do not differ between CDA type II patients with high and low ERFE expression, other yet unknown factors are probably involved in iron homeostasis.

Other very rare types of CDA are caused by autosomal dominant mutations in *KIF23* (Kinesin Family Member 23, CDA type III), *KLF1* (Kruppel-Like Factor 1, CDA type IV) or X-linked mutations in *GATA1* (GATA Binding Protein 1, X-linked thrombocytopenia with or without dyserythropoietic anemia) [56,57,58]. It has been described that in patients with CDA type III, serum iron, ferritin and transferrin levels are generally normal [72]. Interestingly, since hemosiderin staining in urine was positive in most patients with CDA type III, it was hypothesized that this urinary iron loss prevents iron loading. For CDA type IV, less than 10 cases have been described in the literature [73,74,75]. The majority has received transfusions on an occasional or frequent basis, leading to transfusional IO. However, in one pediatric case, hyperferritinemia (>500 ng/mL) and hepatic iron loading (LIC-value of 6.6 mg/g) were observed, while the patient only having received two transfusions throughout the last 5 years [73]. Therefore, besides transfusional iron loading, ineffective erythropoiesis in CDA type IV may disturb iron homeostasis, similarly to in CDA type II or NTDT. There is no data on iron status in X-linked thrombocytopenia with dyserythropoietic anemia [58,76,77]. 

Sideroblastic anemias (SA) represent a group of inherited and acquired disorders characterized by iron accumulation in the mitochondria of erythroid precursors, leading to an abrogated maturation of erythroblasts. Congenital sideroblastic anemia (CSA) is most frequently caused by X-linked mutations in *ALAS2* (Delta-aminolevulinate synthase 2, XLSA), followed by *ABCB7* (ATP Binding Cassette Subfamily B Member 7), *SLC25A28* (Solute Carrier Family 25 Member 28) and *GLRX5* (Glutaredoxin 5). These genes are involved in heme biosynthesis, iron-sulfur cluster biosynthesis and mitochondrial protein synthesis [78]. Whereas systemic IO is common in CSA, and was already reported more than 30 years ago in CSA, the underlying mechanisms have not been studied in much detail [79]. A recent small cases series demonstrated that in patients with CSA, eight out of 13 (8/13, 62%) subjects had LIC-values above 5 mg/g (range 6.18–19.6) with the highest value of ferritin during treatment being above 500 ng/mL in all patients (range of 616–3430). Moreover, significant cardiac IO was found in two out of six (2/6) patients that were analyzed. Although most of these patients had occasional or regular transfusions, significant hepatic IO was also found in a non-transfused patients [80]. While IO in CSA generally develops in the third or fourth decade, it can also present at a very young age, demonstrating the clinical heterogeneity of the disease, even within families [81]. The role of key regulators in ineffective erythropoiesis and iron metabolism has not been studied in much detail in SA. In comparison, in patients with refractory anemia with ring sideroblasts (RARS), an acquired form of SA, it has been demonstrated that GDF-15 levels were substantially increased. Furthermore, mean hepcidin levels were lower in patients with RARS compared to other subtypes of myelodysplastic syndrome (MDS), while mean levels of TSAT and NTBI were increased [82,83,84]. Although a decreased hepcidin/ferritin ratio was measured in a single patient with XLSA, no other studies in CSA have reported hepcidin levels so far. [85] Moreover, intracellular iron homeostasis may be affected due to the specific mutations in CSA. [86] Based on the underlying mechanism of anemia and studies in RARS, we speculate that EPO levels are increased, stimulating erythroid hyperplasia in the bone marrow, resulting in increased ERFE levels. There is no data on sTfR and GDF15 levels in CSA; however, reticulocyte levels are generally within the normal range, despite high EPO levels. This suggests a certain degree of ineffective erythropoiesis occurring within CSA. Moreover, besides ERFE-induced hepcidin suppression, other disease-specific factors may be involved in iron loading in CSA.

Dehydrated Hereditary Stomatocytosis (DHS)/Hereditary Xerocytosis (HX) is characterized by increased cation permeability in the red cell membrane, resulting in abnormal hydration, morphological changes and chronic hemolysis [87]. DHS/XS is caused by autosomal dominant gain-of-function mutations in the *PIEZO1* gene (PIEZO-Type Mechanosensitive Ion Channel Component 1, DHS 1) or *KCCN4* gene (Potassium Channel Calcium-activated Intermediate/Small Conductance Subfamily N Member or Gardos channel, DHS 2) [88,89]. Interestingly, despite adequate compensation of hemolysis and in general little need for transfusions, IO is common in both DHS, illustrated by high ferritin levels in 36% of patients (>500 ng/mL) and mean LIC levels of 11 mg/g at diagnosis. Whereas there was a clear positive correlation between ferritin levels >1000 ng/mL and LIC, this could not be found when ferritin levels were <1000 ng/mL, suggesting that also in DHS, ferritin levels cannot predict the presence of significant IO [90]. A recent study demonstrated that hepcidin levels are suppressed in patients with *PIEZO1*-mutated DHS, while ERFE levels were only slightly elevated, suggesting an additional regulatory mechanism of hepcidin suppression and subsequent iron loading in DHS [91,92]. It was shown that increased Ca^2+^ influx through the mutated *PIEZO1* channel directly decreased *HAMP* gene expression [91]. While very little is known about the regulation of iron loading in Gardos channelopathy specifically, it has been suggested that this is similar to *PIEZO1* DHS. In conclusion, iron loading is common in DHS. Besides ERFE-induced hepcidin suppression, mutations in *PIEZO1*, and possibly in *KCCN4*, directly alter iron metabolism, resulting in hepcidin suppression.

In Sickle Cell Disease (SCD), caused by biallelic specific amino acid changes (Glu6Val, HbSS) or compound heterozygous mutations including hemoglobin S (HbS) combined with hemoglobin C (HbSC) or beta-thalassemia (HbS/beta-thalassemia), the formation of hemoglobin S (HbS) causes RBCs to sickle under certain conditions, leading to vaso-occlusion, inflammation and hemolysis [93,94]. Interestingly, in SCD both transfused and non-transfused patients have a lower risk of developing IO [95]. Although the underlying mechanisms have not been studied extensively, this has historically been explained by less transfusional IO in the majority of patients and the chronic inflammation in SCD. The latter has been associated with elevated levels of IL-6, a positive regulator of hepcidin expression. As for erythropoiesis, both EPO and sTfR levels are increased and GDF15 levels may be mildly elevated, illustrating increased erythroid drive and possible ineffective erythropoiesis [21,31,42]. Hepcidin levels in SCD positively correlated with ferritin levels, although hepcidin/ferritin ratios were lower than in healthy controls [96,97,98]. There is conflicting evidence on the correlations between hepcidin and both inflammation and erythroid drive, illustrating the complex interplay of factors which may occur uniquely in each individual patient [96,99,100,101]. ERFE levels in SCD are elevated, although less evident than in PKD and NTDT [42,98]. Interestingly, there was no significant correlation found between hepcidin and ERFE. Altogether, regulation of iron metabolism in SCD is multifactorial and potential protectors against IO have not been determined yet. However, it is conceivable that in SCD, ERFE mediated hepcidin suppression might be partially counteracted by the IL-6 mediated hepcidin induction, leading to a lower tendency to develop iron loading in SCD compared to NTDT. In addition, patients with SCD can have significant levels of hemoglobinuria following intravascular hemolysis, which contributes to SCD-related renal disease, but also results in urinary iron loss [102,103]. 

Diamond-Blackfan anemia (DBA) is an inherited bone marrow failure syndrome which is characterized by hypoplastic anemia (reticulocytopenia), congenital malformations and cancer predisposition [104]. Since DBA is predominantly caused by mutations in genes encoding for ribosomal proteins, it is classified as a ribosomopathy. In DBA, approximately 30% of patients are treated with chronic transfusions, whereas 35-40% of patients respond to steroids, which until now have been the only drug proven to be effective for DBA. The remainder are either treatment-independent or have been treated with allogeneic stem cell transplantation [104,105]. While IO is a serious problem in patients that have been transfused since a young age, there is little knowledge concerning iron status in steroid-dependent or treatment-independent patients [106] In a small case-study of four patients treated with glucocorticoids and seven patients in disease remission, iron status was evaluated, demonstrating mean TSAT levels of 64% and 38%, and serum ferritin levels ranging from 44–637 ng/mL (mean 188 ng/mL) and 42–1079 ng/mL (mean 177 ng/mL) respectively [107]. These data suggest that IO may also occur in non-transfusion-dependent DBA patients. Interestingly, it has been shown that the distribution of iron in DBA differs from other RHA. In a study comparing IO (defined by serum ferritin >1500 ng/mL or LIC >7 mg/g) in transfusion-dependent patients with DBA, SCD or beta-thalassemia, the levels of NTBI were higher in DBA than in both beta-thalassemia and SCD (2.50 µmol/L versus 1.68 and −0.23, respectively) [108] The levels of sTfR were undetectable in DBA patients, illustrating the decreased erythroblast maturation, and hepcidin levels were appropriately elevated. This suggests that iron loading in transfusion-dependent DBA differs from beta-thalassemia, possibly as a result of impaired iron uptake and utilization in erythroid precursors, resulting in an elevated NTBI. Second, in a cohort of transfusion-dependent patients (consisting of SCD, TDT, DBA, PKD, CDA patients), it was demonstrated that DBA patients overall had the highest level of organ IO, especially pancreatic and cardiac iron loading [109]. Together, these studies demonstrate that iron homeostasis in DBA is differently regulated than other RHA, including in transfusion-dependent patients. It is known that DBA is characterized by extremely high EPO levels accompanied by relative EPO sensitivity [108,110]. Moreover, GDF15 levels are mildly elevated in DBA and tend to be higher in patients treated with glucocorticoids compared to transfusion-dependent patients, and sTfR levels can also be detected in glucocorticoid responsive patients [107,108]. Hypothetically, in DBA patients that are responsive to glucocorticoid treatment, ERFE levels may be elevated and these patients may be at risk for non-transfusional IO. Overall, iron metabolism in DBA is poorly understood, indicating that in both tranfusion-dependent and non-transfusion-dependent regular evaluation of organ IO is necessary. 

Hereditary spherocytosis (HS) is a relatively common hereditary hemolytic anemia caused by defects in the cellular membrane of RBCs [111] The majority of defects occur in five different genes encoding red blood cell membrane proteins: *SPTA1* (alpha-spectrin), *SPTB* (beta-spectrin), *ANK1* (Ankyrin 1), *SLC4A1* (Band 3 or solute Carrier Family 4) and *EPB42* (Erythrocytic Protein 4.2) [112,113]. IO is uncommon in HS, yet has been described with a co-inheritance of HH [114,115]. In general, patients with HS have increased reticulocyte counts and elevated EPO levels, to compensate for anemia. In the majority of patients, anemia is relatively mild as a result of effective compensation. In patients that suffer from severe anemia, or other complications of HS, such as splenomegaly and hyperbilirubinemia, there is an indication for splenectomy, which results in a substantial decrease in chronic hemolysis and improvement of the clinical phenotype in most patients [116]. Interestingly, old histopathological studies of spleens of HS patients have demonstrated moderate to severe iron accumulation [117]. Splenic iron accumulation as well as the rapid recycling of iron in erythropoiesis could explain why systemic IO is rarely seen in severe non-splenectomized HS patients. Additionally, we speculate that alleviation of the anemia by performing a splenectomy could thus also normalize the iron metabolism. However, recently it has been described in four adults with mild HS (Hb 110–122 g/L and reticulocyte counts of 12.6–17%) that IO can occur, illustrated by severe hepatic IO in two patients and elevated LIC in two other patients (LIC 6.6 and 6.9 mg/g) [118]. Genetic testing in these patients excluded HH as a cause of iron loading. This suggests that mild HS can in some cases be accompanied by IO, yet the underlying mechanism has not been studied in detail. Theoretically, the patients most prone to develop IO are patients that have uncompensated anemia, since these patients have reticulocytosis and persistently elevated EPO levels. A recent report showed that mean ERFE levels in twenty-four adult patients with HS (10/24 post-splenectomy) were slightly elevated compared to healthy controls (4 versus 2 ng/mL), while the variation was high (2–22 ng/mL), accompanied by mean EPO levels of 14 IU/L (6–31 IU/L), and normal iron parameters [42]. Altogether, this suggests that IO is rare in HS and is most likely related to negligible ineffective erythropoiesis, yet can occur in selected patient groups. Therefore, this rare mechanism needs to be studied in more detail in order to correctly identify which patients are at risk for IO. 

## 3. Discussion

Here, we have given an overview of important regulators of iron metabolism in the context of RHA (summarized in Table 2). Whereas in most RHA, increased erythropoietic drive in combination with ineffective erythropoiesis stimulate iron overload, in SCD and DBA other mechanisms appear to play a role. In SCD, this might be due to chronic inflammation inducing hepcidin expression and counteracting ERFE-induced inhibition of hepcidin [96,98,101]. In agreement with this, recent data have demonstrated that, while erythroid output is increased in SCD, ERFE levels are relatively low, especially when compared to patients with PKD and beta-thalassemia [42]. As a consequence, in patients with SCD, not treated with chronic transfusions, this leads to iron restriction instead of IO which may improve SCD-related outcomes [119]. While in DBA increased hepcidin levels theoretically protect against pathological iron absorption, this also impedes iron availability, thereby further limiting RBC production. However, since in DBA there is disturbed balance between heme and globin production, relative iron restriction could also be protective, reducing the amount of toxic free heme that worsens erythroid development in DBA [120,121].

In addition to the key regulators discussed here, other regulators of iron metabolism may provide important links in understanding iron loading in the subtypes of RHA. It has been demonstrated that chronic EPO administration in both ERFE-KO and wild-type mice resulted in similar hepcidin suppression, suggesting that, although ERFE is an important regulator of iron homeostasis upon acute erythroid stimulation, it may be dispensable during chronic erythroid drive [123]. Soluble hemojuvelin (sHJV), a glycophosphatidylinositol (GPI)-linked membrane protein which regulates hepcidin expression through inhibition of the BMP/SMAD pathway, has been thoroughly studied as an iron regulator that arises in hypoxic conditions [124]. Little is known concerning the role of sHJV in RHA. In CDA type I and NTDT, it has been demonstrated that sHJV levels are increased compared to healthy controls and it is conceivable that this also occurs in other RHA [63,125]. Another candidate regulator of iron metabolism in hypoxic conditions is platelet-derived growth factor-BB (PDGF-BB), a growth factor involved in cellular growth and proliferation [126]. While hypoxia-induced PDGF-BB upregulation leads to downregulation of hepcidin expression, PDGF-BB levels do not respond to EPO treatment in mice, illustrating that PDGF-BB expression occurs upon hypoxia without the interference of EPO. This suggests that PDGF-BB regulates hepcidin expression independently of erythropoietic activity [127,128]. In the gut, hypoxia inducible factor-2α (HIF-2α), a protein essential for adaptation during hypoxia, is stabilized in enterocytes upon low oxygen levels. HIF-2a induces transcription of DMT1 and as a result increases enteral iron absorption [129]. Since in iron-deficiency, HIF-2a can induce upregulation of ferroportin in enterocytes, this might also occur in hypoxic conditions, facilitating the secretion of the absorbed iron to the bloodstream [130]. In addition to GDF15, another member of the TGFß superfamily of proteins, growth differentiation factor 11 (GDF11), has been studied in the context of RHA. GDF11 is expressed in various cell types, including immature erythroid progenitors, and has been functionally linked to progenitor expansion at the expense of erythroid differentiation [131]. The new therapeutic luspatercept, which acts as a trap ligand to inactivate GDF11, has been shown to increase RBC count and hematocrit in healthy and anemic individuals [132,133]. While it has been demonstrated that GDF11 can inhibit hepcidin levels, thereby connecting erythropoiesis to iron metabolism, other studies have questioned the role of GDF11, since the murine homolog of luspatercept, RAP-536, also enhanced erythropoiesis in GDF11 KO mice [134,135]. Therefore, further studies are needed to investigate the role of GDF11 in human erythropoiesis and iron regulation. Based on previous studies in beta-thalassemia, Twisted Gastrulation BMP Signaling Modulator (TSWG1), produced by erythroblasts was identified as a candidate regulator protein of erythropoiesis and iron metabolism, yet its physiological role was not confirmed in follow-up studies [128,136,137]. 

In summary, we have illustrated that IO is common in a wide range of mechanistically distinct forms of RHA, predominantly mediated by drivers of (ineffective) erythropoiesis and hepcidin expression. By determining the rate of erythropoietic drive and degree of ineffective erythropoiesis, the risk of IO could be estimated. At the same time, the knowledge of specific regulators in distinct disease entities, and the direct effect of IO on the quality of erythropoiesis is still largely unknown and primarily based on relatively small case series. In order to improve our understanding of the interplay between erythropoiesis and IO, international consensus guidelines for the screening, investigating and monitoring of IO in combination with uniformal registration of patients with RHA are a prerequisite. 

## Figures and Tables

**Table 1:** Overview of diagnostic tools in assessment of iron overload. Iron overload (IO), liver iron content (LIC), magnetic resonance imaging (MRI), non-transferrin bound iron (NTBI), rare hereditary anemia (RHA), Transferrin saturation (TSAT).

Test	Cut-Off Values	Advantages	Disadvantages
Serum ferritin	>500 μg/L (for chelation >800μg/L) [17]	Widely available. Inexpensive.	Does not correlate with severity of IO in RHA. Is elevated upon inflammation.
TSAT	>60%	Possible in most health centers. Closer relation to extrahepatic IO.	Cannot be interpreted correctly during iron chelation therapy.
Liver biopsy	>3 mg/g	Golden-standard for liver IO.	Invasive. Dependent on tissue handling.
MRI	LIC >3 mg/g Cardiac T2* <20ms	Not invasive.	Expensive. Not widely available.
NTBI	>0	Relevant (relates closely to organ damage).	Not widely available. High interlaboratory variation. Is decreased upon inflammation.

**Table 2:** Overview of proposed mechanisms in the various types of rare hereditary anemia. CDA congenital dyserythropoietic anemia; CSA congenital sideroblastic anemia; DBA Diamond-Blackfan anemia; DHS dehydrated hereditary stomatocytosis; EPO erythropoietin; GDF-15 growth differentation factor 15; HS hereditary spherocytosis; IO iron overload; NTDT non-transfusion-dependent thalassemia; PKD pyruvate kinase deficiency; sTfR soluble transferrin receptor.

Mechanism		Prevalence of IO	EPO	sTfR	GDF-15	Hepcidin or Hepcidin/Ferritin Ratio ***	Erythroferrone *	Summary
**Both ineffective erythropoiesis and hemolysis**	NTDT	Very common	Extremely high	Increased	Extremely high	Extremely low	Extremely high	Increased iron loading due to ERFE-induced hepcidin suppression as a result of ineffective erythropoiesis.
	PKD	Very common	Increased	Presumably increased	Increased	Decreased	Increased	Increased iron loading due to ERFE-induced hepcidin suppression as a result of ineffective erythropoiesis, yet to a lesser extent than in NTDT.
	HbH disease	Common	Increased	Increased	Increased	Decreased	Presumably increased	Risk of increased iron loading, presumably due to ERFE-induced hepcidin suppression as a result of increased/ineffective erythropoiesis.
	CDA type II	Common	Increased	Increased	Normal—increased	Decreased - normal	Extremely high	Increased iron loading due to ERFE-induced hepcidin suppression as a result of ineffective erythropoiesis, yet to a lesser extent than in NTDT. Other yet unknown factors may play a role.
	SCD	Uncommon	Increased	Increased	Increased	Variable	Increased	Iron loading is less profound. Altered iron metabolism due to ERFE-induction as a result of increased/ineffective erythropoiesis, simultaneous to increased hepcidin expression due to inflammation.
**Reduced or ineffective erythropoiesis**	CDA type I	Common	Increased	Increased	Extremely high	Decreased—normal	Presumably increased	Risk of increased iron loading, presumably due to ERFE-induced hepcidin suppression as a result of increased/ineffective erythropoiesis. Other yet unknown factors may play a role.
	DBA	Common	Very high **	Decreased—normal	Variable	Variable	Presumably decreased—normal	Iron loading is less profound. Altered iron metabolism which is poorly understood. Individual assessment of iron status is necessary.
	CSA	Common	Increased	Presumably increased	Presumably increased	Presumably decreased	Presumably increased	Risk of increased iron loading, presumably due to ERFE-induced hepcidin suppression as a result of ineffective erythropoiesis. Other yet unknown factors may play a role.
**Hemolytic anemia**	HS	Uncommon	Normal—increased	Normal—increased	Normal—increased	Decreased—normal	Normal—increased	Iron loading is rare. In specific patient groups with continuous hemolysis and increased/ineffective erythropoiesis, iron loading may occur due to hepcidin suppression.
	DHS	Common	Increased	Increased	Normal—increased	Decreased	Increased	Risk of increased iron loading, presumably due to ERFE-induced hepcidin suppression as a result of increased/ineffective erythropoiesis. Other factors may play a role, such as PIEZO1-dependent hepcidin suppression.

Mean EPO range: normal 0–20 IU/L; increased 20–40 IU/L; extremely high >40 IU/L; Mean sTfR range: decreased <2 mg/L; normal 2–5 mg/L; increased; Mean GDF - 15 range: normal 200–900 pg/mL; increased 900–10,000 pg/mL; extremely high >10,000 pg/mL; Mean Hepcidin or hepcidin/ferritin ratio range ***: n.a.; Mean ERFE range: * decreased < 2 ng/mL; normal 0–2 ng/mL; increased 2–20; extremely high >20 ng/mL. * = No standardized references values are available for ERFE, these cut-off values are based on recent studies [39,68]. ** = In addition to consistently high EPO levels, EPO-sensitivity is decreased. *** = For hepcidin analysis, various methods have been used in the past decade. In order to improve the diagnostic value of hepicidin analysis and compare between studies, hepcidin/ferritin levels have been used [122].

## Data Availability

Not applicable.
